# Lifestyle habits, macronutrient intake, and obesity prevalence among adolescents in rural-periurban community senior high schools in the Ho municipality of Ghana

**DOI:** 10.3389/fnut.2022.955898

**Published:** 2022-08-30

**Authors:** Sheila Akoto, Marina Aferiba Tandoh, Kwabena Nsiah, Odeafo Asamoah-Boakye, Veronica Tawiah Annaful

**Affiliations:** Department of Biochemistry and Biotechnology, Faculty of Biosciences, College of Science, Kwame Nkrumah University of Science and Technology, Kumasi, Ghana

**Keywords:** lifestyle habits, macronutrient intake, obesity prevalence, adolescents, senior high schools

## Abstract

**Background:**

Adolescence is a critical stage in the life cycle that presents a window of opportunity for the formation of lifetime habits or an aversion to childhood malnutrition effects. This study assessed the lifestyle habits, macronutrient intakes, and obesity prevalence among adolescents in some selected Senior High Schools in rural communities in Ho Municipality.

**Materials and methods:**

A cross-sectional survey was conducted among 272 adolescents aged 13–19 years and attending senior high schools in the Ho Municipality of Ghana. Data on sociodemographic, physical activity levels, dietary habits, and anthropometrics were obtained. A body mass index (BMI) and waist circumference (WC) were determined, while a repeated 24-h dietary recall was used to collect the dietary intakes of the participants.

**Results:**

The majority of the adolescents did not meet the Recommended Dietary Allowances (RDA) for calories (94.5%), dietary protein (68.8%), and fibre (91.5%). Adolescent boys consumed more calories (1,969.7 ± 579.9 Kcal) on average than adolescent girls (1,658.0 ± 458.7 Kcal) (*p* = 0.001). Overweight and obesity prevalence were 15.8 and 8.5%, respectively. About 90.4% of the adolescents did not meet the WHO recommended 150 min per week of physical exercise. On sedentary, 97.6% of adolescents spent half an hour to 5 h per day watching television when at home. Breakfast was the most frequently skipped meal (47.9%), and 59.6% of adolescents consumed fast foods such as pizza, burgers, and ice cream one to three times per week. Adolescent girls also had higher odds of being overweight or obese compared with adolescent boys (AOR = 2.4, *p* = 0.094, 95% CI = 0.9–6.4). Adolescents who did not meet the RDA for calories had lower odds of being overweight or obese compared with those who did (UOR = 0.3, *p* = 0.045, 95% CI = 0.1–0.9).

**Conclusion:**

Poor dietary habits and intake, sedentary lifestyle, and obesity prevalence were observed among the adolescents. Being an adolescent girl was associated with obesity risk, while not meeting caloric intake showed a protective effect. Efficient and effective nutrition and lifestyle education programme should be promoted in communities to improve the dietary intake and lifestyle habits of adolescents.

## Introduction

The incidence of obesity has tripled in developing countries over the past three decades (1) with increasing urbanization, flexible trade policies, globalization, and a massive increase in food processing companies (2). While there has been an 18.3% reduction in underweight among adolescent students in Ghana between 2007 and 2015, there has been a rise in overweight and obesity from 8.7% in 2007 to 13% in 2015 (3, 4). Overweight and obesity are important risk factors for non-communicable diseases (NCDs) such as cardiovascular diseases, cancers, chronic respiratory diseases, and diabetes (5). In addition, obesity in adolescents has been associated with high sedentary behaviours (6), physical inactivity, long screen times, and poor dietary behaviours (7) in different settings.

Due to globalization and the availability of fast-foods, dietary intakes in Ghana have shifted from consumption of wholegrain, non-refined carbohydrate intake in the past to a significant amount of highly processed foods and fast foods such as pastries, pizza, and sugar-sweetened beverages (8, 9). Severe food insecurity has also contributed to fast food consumption among adolescents in low-income countries (10). This is because people who are severely hungry may prefer energy-dense foods to compensate for times when food is scarce (10, 11). The consumption of sugar-sweetened beverages and highly processed foods, which contain few micronutrients but are high in calories, sugar, saturated and trans-fat, has been the driver of increasing rates of obesity among adolescents in low-and-middle-income countries (12). In Ghana, it has been reported that the dietary patterns of schooling adolescents are predominantly sugar-sweetened snacks, energy drinks, and soft drinks (8). Prospective cohort studies in children and adolescents also suggest that intake of highly processed foods contributes to high levels of body fat and obesity (13–15). Other dietary behaviour such as skipping breakfast and low dietary fibre intake have also been reported among adolescents in Ghana (8, 16), and may also contribute to the increasing obesity in this population (17–19). Furthermore, inadequate nutrient intake has also been found among schooling adolescents in Ghana (20).

In addition, low physical activity and sedentary behaviours are increasing among children and adolescents (21). For adolescents, early exposure to unhealthy lifestyle behaviours increases their risk of NCDs in adulthood. The United Nations predicts that within the next few decades, Africa and Asia will have the largest and fastest urban population growth (22). High population densities in cities, particularly in low-and middle-income countries, often increase sedentary lifestyles (21, 23). In addition, urbanization, coupled with an influx in motorized transportation use in densely populated cities (24) may reduce physical activity participation (25). Available data shows that 75% of adolescents aged 13–18 years in Ghana are physically inactive (26). Sedentary lifestyles such as watching television, using the computer, the internet, and social media are also increasing among adolescents in Ghana (27). Inadequate physical activity and sedentary behaviours may also contribute to increasing obesity among children and adolescents (27).

For children and adolescents, the WHO (28) identifies several strategies to prevent the insurgence of adolescent obesity, including promoting an active lifestyle, reducing television viewing, promoting fruit and vegetable intake, creating more opportunities for family interaction (e.g., eating family meals), restricting the intake of energy-dense, micronutrient-poor foods (e.g., packaged snacks) and the intake of sugar-sweetened soft drinks. As adolescents spend the most time in school and become more independent in their food choices, the school environment becomes an important place in shaping dietary behaviours (8). It may present an opportunity to encourage physical activity and steer the diet of adolescents toward healthier options (29), or lead to poor dietary habits (30). Identifying lifestyle habits, macronutrient intake and obesity prevalence among adolescents could be important to understand the lifestyle and dietary behaviours of adolescents and inform intervention for improved dietary and lifestyle practice.

## Materials and methods

### Study design and population

The study adopted a cross-sectional design. The study population comprised 272 adolescents aged 13–19 years attending Senior High Schools (SHS) in the Ho Municipality of Ghana. The study population was male and female schooling adolescents who either lived in the Ho municipality or stayed in the municipality for school. The study population was either day-going students who lived in the Ho communities and attend school during the day or boarding students who stayed in the school until vacation.

### Ethics

The study was approved by the Committee on Human Research Publication and Ethics, of the School of Medicine and Dentistry, Kwame Nkrumah University of Science and Technology, Kumasi (CHRPE/AP/026/20). Permission letters to carry out the study were also obtained from the Head Teachers of the selected Senior High Schools. Participation in this study was voluntary. Written informed consent and assent were obtained from parents or guardians and students below the age of 18 years and below, respectively, before enrolling them in the study. Similarly, written informed consent was obtained from the adolescents aged 18–19 years before being recruited to participate in the study. The study included adolescent students who were physically healthy, had attended school for at least one academic year, and agreed to participate.

### Sample size determination

The sample size of the study was determined using the Cochran formula. The formula is stated as follows:


$$n_{0}=\frac{Z^{2}\,p\,q}{e^{2}}$$

Where *e* is the desired level of precision (i.e., the margin of error) = (5%),

*p* is the (estimate) proportion of the population which has the attribute in question = 50%,

*q* is 1-p.

*Z*^2^ is the abscissa of the normal curve that cuts off an area at the tails. The value of *Z* is found at a 95% confidence level is 1.96 (*Z* 1.96) as found in the statistical table.

The sample size is calculated as:


$$n_{0}=\frac{1.96^{2}*\left(0.5\right)\,\left(0.5\right)}{0.05^{2}}$$

An estimated sample size of 305 participants was the target population, however, we obtained 282 participants due to time constraints and the global pandemic that caused restrictions.

### Data sampling

The study used both stratified and simple random sampling approaches to recruit participants from eight (8) senior high schools. Ten SHS in the Ho Municipality were deemed appropriate for the study. First, the SHS was stratified into government-owned and private-owned senior high schools. In each stratum, numbers were given to each school on pieces of paper. The papers were folded and placed in opaque boxes according to each stratum. Five SHS each from each stratum were randomly drawn from the opaque boxes. However, only three (3) private SHS responded to our research invitation, so we had eight SHS participating in the study. Using convenient sampling, we aimed to select 40 students from each SHS. However, in some schools, we fell short of the target number of participants, resulting in 282 participants in all.

### Data collection

A questionnaire was developed and used to assess the gender, age, physical activity levels, and dietary habits of participants. Other sociodemographic characteristics of the participants were obtained but are not reported in this manuscript. A repeated 24-h dietary recall on two consecutive weekdays and one weekend was used to assess the nutrient intakes of the participants. We took weight (in kg), height (in centimetres), and waist circumference measurements. Day training workshop was organized for all enumerators to confidently equip themselves with anthropometrics while MPhil Human Nutrition and Dietetics students collected the diet history.

#### Anthropometric measurement

All anthropometric indices were carried out following WHO standardized protocols. The weighing scale and stadiometer were placed on a flat and smooth surface to ensure accuracy in measurements. The heights of the participants were measured using a stand-alone stadiometer (SECA 213, Hamburg, Germany). The students were asked to stand straight on the stadiometer with their heads, buttocks, and heels touching the pole of the stadiometer. Readings were taken at eye level to ensure the correct measurement and were taken to the nearest 0.1 cm. These measurements were taken twice, and the average was recorded.

A weighing scale (Omron BF211, Tokyo, Japan) was used to determine the weights of the participants, in light garments and with no shoes on. They were asked to stand in the centre of the platform such that their weight was evenly distributed to both feet. The weight was measured twice, and the average was taken to the nearest 0.1 kg. Body mass index (BMI) was calculated using weight divided by height squared, and classification was based on WHO cut-offs (31) (i.e., underweight, normal, overweight, and obese). A BMI ≥ 25.0 kg/m^2^ was considered overweight or obese.

Waist circumference (WC) was measured using an inextensible measuring tape. The tape was placed at the midpoint between the lower margin of the last palpable rib and the top of the iliac crest according to WHO protocol (32).

#### Dietary intake assessment

A repeated 24-h dietary recall on two weekdays and one weekend was used to obtain the dietary intake of the participants. The first 24 h recall was taken from the previous day’s dietary intake. The recalls were conducted *via* face-to-face interviews by trained MPhil Human Nutrition and Dietetics students, who were also enumerators. The portion sizes of the adolescent students’ food intake were estimated using household food measures. The portion sizes in household food measures were converted into their gram equivalents (33). The trained MPhil Human Nutrition and Dietetics students recorded grams of the foods consumed into a nutrient analysis template designed by the University of Ghana, Department of Food Science and Nutrition (Accra, Ghana) (34), which contains macronutrient and micronutrient levels in most Ghanaian foods. The nutrients from the 3-day dietary recall were obtained and an average of the 3 recalls was calculated. We compared the mean macronutrients obtained to the Recommended Dietary Allowance (RDA) of the same age group using the National Academy of Sciences, Food and Nutrition Board cut-offs (35). Mean calories were compared to estimated energy requirement (EER) using the National Academy of Sciences, Food and Nutrition Board cut-offs (35). Adequate nutrient intakes were categorized as “met RDA” and inadequate intakes were categorized as “not met RDA.”

### Data analysis

The Statistical Package for Social Sciences version 26 (SPSS; IBM Inc., Chicago, IL, United States) was used to analyse the data. A test of normality was performed on the anthropometric measurements and nutrient intakes. Descriptive statistics were performed on gender, age group, dietary habits, physical activity levels, anthropometric indices, and nutrient intake categories. Crosstabulation tests were used to compare the frequencies of nutrient intake status, BMI status by gender, and age group of participants. Similarly, a chi-square (Fisher’s exact test) crosstabulation test was performed to compare the frequencies of dietary habits, physical activity levels, nutrient intakes, and BMI status. An independent *t*-test was used to compare the mean difference between BMI, WC, and nutrient intakes by gender and age group, and a significant difference was determined using the Mann–Whitney “U” test for non-parametric comparison. An analysis of variance (ANOVA) analysis was performed to compare the mean difference between waist circumference, nutrient intake, and BMI status of participants. The significant difference was determined using the Kruskal–Wallis test for non-parametric comparison. Data for mean comparison were presented as mean ± SD (standard deviation). A Pearson correlation controlling for gender and age was performed to determine the association between WC, BMI and nutrient intakes. Unadjusted and adjusted (for age) binary logistic regression tests were performed to determine predictors of overweight/obesity. All statistical tests were two-tailed, with *p*-values less than 0.05 considered significant.

## Results

**FIGURE 1:**
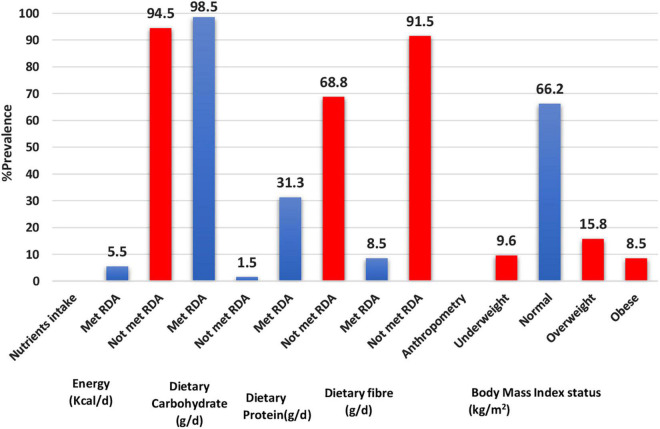
Adequacies and inadequacies in calories and macronutrient intakes, and BMI status.

Figure 1 presents adequacies and inadequacies in calories and macronutrient intakes, and BMI status. The majority of the adolescents did not meet RDA for calories (94.5%), dietary protein (68.8%), and dietary fibre (91.5%). Obesity prevalence was 8.5%, while 15.8% of adolescents were overweight.

Table 1 presents the relationship between gender, age group, calorie and macronutrient intakes, and anthropometric variables. Adolescent boys (15.0%) met the RDA for dietary fibre non-significantly more than adolescent girls (7.3%). More 18–19 years old adolescents (6.8%) met the RDA for calories (*p* = 0.432) than 13–17-year-old adolescents (4.5%). Adolescent boys (1969.7 ± 579.9 Kcal) consumed more mean dietary calories than adolescent girls on average (1658.0 ± 458.7 Kcal) (*p* = 0.001). Adolescent boys consumed more mean dietary carbohydrates (310.6 ± 92.6 g) than adolescent girls (264.9 ± 70.9 g) (*p* = 0.005). Similarly, adolescent boys consumed more mean dietary protein (51.2 ± 17.7 g) than adolescent girls (41.6 ± 10.4 g) (*p* = 0.001). Adolescent boys had higher mean dietary fat (61.3 ± 20.0 g) than adolescent girls (50.0 ± 20.6 g) (*p* < 0.001). Also, adolescent boys had higher mean dietary fibre (24.4 ± 8.9 g) than adolescent girls (17.8 ± 5.3 g) (*p* < 0.001). Adolescents aged 18–19 years consumed more mean dietary fibre (53.9 ± 22.9 g) than adolescents aged 13–17 years (49.9 ± 19.2 g) (*p* = 0.047).

**TABLE 1:** Sociodemographic variables, calories and macronutrient intakes, and anthropometric variables.

	Gender		*P*-value	Age group (years)		*P*-value
				
Variable	Boys	Girls		13–17 years	18–19 years	
Nutrient intakes	*N* = 40	*N* = 232		*N* = 155	*N* = 117	
**Calories, Kcal**						
Met RDA	2 (5.0)	13 (5.6)	1.000^¥^	7 (4.5)	8 (6.8)	0.432^¥^
Not met RDA	38 (95.0)	219 (94.4)		148 (95.5)	109 (93.2)	
Mean calories	1969.7 ± 579.9	1658.0 ± 458.7	0.001^†^	1670.6 ± 461.9	1747.8 ± 523.3	0.243**^†^**
**Dietary carbohydrate, g**						
Met RDA	40 (100.0)	228 (98.3)	1.000^¥^	153 (98.7)	115 (98.3)	1.000^¥^
Not met RDA	0 (0.0)	4 (1.7)		2 (1.3)	2 (1.7)	
Mean dietary carbohydrate	310.6 ± 92.6	264.9 ± 70.9	**0.005^†^**	267.7 ± 72.3	276.9 ± 80.7	0.348**^†^**
**Dietary protein, g**						
Met RDA	12 (30.0)	73 (31.5)	1.000^¥^	49 (31.6)	36 (30.8)	0.896^¥^
Not met RDA	28 (70.0)	159 (68.5)		106 (68.4)	81 (69.2)	
Mean dietary protein, g	51.2 ± 17.7	41.6 ± 10.4	**0.001^†^**	42.3 ± 11.5	43.9 ± 13.1	0.379**^†^**
**Dietary fibre, g**						
Met RDA	6 (15.0)	17 (7.3)	0.123^¥^	14 (9.0)	9 (7.7)	0.827^¥^
Not met RDA	34 (85.0)	215 (92.7)		141 (91.0)	108 (92.3)	
Mean dietary fibre	24.4 ± 8.9	17.8 ± 5.3	**<0.001^†^**	49.9 ± 19.2	53.9 ± 22.9	**0.047^†^**
Mean dietary fat, g	61.3 ± 20.0	50.0 ± 20.6	**<0.001^†^**	18.2 ± 6.3	19.6 ± 6.5	0.205**^†^**
**Anthropometry**						
Mean waist circumference, cm	74.5 ± 5.5	73.3 ± 10.7	0.079**^†^**	73.1 ± 12.0	74.0 ± 6.7	**0.028^†^**
**BMI, Kg/m^2^**						
Underweight	3 (7.5)	23 (9.9)	0.120^¥^	97 (62.6)	83 (70.9)	0.339^¥^
Normal	32 (80.0)	148 (63.8)		41 (26.4)	25 (21.4)	
Overweight/Obese	5 (12.5)	61 (26.3)		17 (11.0)	9 (7.7)	
Mean BMI	21.6 ± 2.9	23.3 ± 4.5	**0.031^†^**	23.1 ± 4.8	23.0 ± 3.8	0.776**^†^**

Mean ± SD (standard deviation), ^†^Mann Whitney test, ^¥^Fisher’s test *p*-value, RDA, Recommended dietary allowance; BMI, Body Mass Index. Bold values means the *p* values are significant.

Adolescent girls were more likely to be overweight or obese (26.3%) than adolescent boys (12.5%) (*p* = 0.120). Overweight or obese adolescents were more prevalent among 13–17 years old (11.0%) than among 18–19 years old adolescents (7.7%) (*p* = 0.339). The BMI of adolescent girls was higher (23.3 ± 4.5 kg/m^2^) than that of adolescent boys (21.6 ± 2.9 kg/m^2^) (*p* = 0.031). On the average, 18–19-year-old adolescents had higher waist circumference measurements (74.0 ± 6.7 cm) than 13–17-year-old adolescents (73.1 ± 12.0 cm) (*p* = 0.028).

Table 2 indicates the relationship between physical activity participation and body mass index status. About 7 in 10 (70.6%) of the adolescents participated in high-intensity exercises, while 22.8% of the adolescents performed no physical exercise. In the previous week, 54.0% of the adolescents exercised once to three times. The majority of the adolescents (55.9%) participated in 20 min or more of physical exercise in the past week. However, 90.4% of the adolescents did not meet the WHO recommended 150 min per week of physical exercise. In terms of sedentary activity, 61.4% watched television 1–3 times per week when at home, while 97.6% of the adolescents spent half an hour to 5 h per day watching television. Adolescents who did not engage in any physical activity were more likely to be slightly overweight or obese than those who engaged in moderate- to high-intensity physical activity (*p* = 0.755). Adolescents who watched television 1–3 times per week (27.5%) had the highest proportions of overweight or obesity compared with those who watched television every day (20.2%) and those who rarely watched television (22.2%) (*p* = 0.159).

**TABLE 2:** Physical activity participation and body mass index status.

		Body Mass Index status			*P*-value
			
Physical activity level (PA)	Total for PA	Normal	Overweight/obese	Underweight	
Type of physical activity	*N* = 272	*N* = 180	*N* = 66	*N* = 26	
High intensity**^†^**	192 (70.6)	129 (67.2)	43 (22.4)	20 (10.4)	0.755^ⱡ^
Moderate intensity^α^	18 (6.6)	11 (61.1)	5 (27.8)	2 (11.1)	
None	62 (22.8)	40 (64.5)	19 (29.0)	4 (6.5)	
**PA in the past week**					
None	68 (25.0)	43 (63.2)	20 (29.4)	5 (7.4)	0.419^ⱡ^
1–3 day(s)	147 (54.0)	98 (66.7)	31 (21.1)	18 (12.2)	
4–5 days	28 (10.3)	18 (64.3)	7 (25.0)	3 (10.7)	
More than 5 days	29 (10.7)	21 (72.4)	8 (27.6)	0 (0.0)	
**Duration of physical exercise**					
Less than 20 min	120 (44.1)	79 (65.8)	28 (23.3)	13 (10.8)	0.800^¥^
≥20 min	152 (55.9)	101 (66.4)	38 (25.0)	13 (8.6)	
**PA recommendation**					
Met 150 min/week	26 (9.6)	16 (61.5)	9 (34.6)	1 (3.8)	0.310^ⱡ^
Not met 150 min/week	246 (90.4)	164 (66.7)	57 (23.2)	25 (10.2)	
**Home TV watching frequency**					
Never/occasionally	13 (4.8)	8 (61.5)	2 (15.4)	3 (23.1)	0.314^ⱡ^
1–3 times per week	167 (61.4)	109 (65.3)	45 (26.9)	13 (7.8)	
Everyday	92 (33.8)	63 (68.5)	19 (20.7)	10 (10.9)	
**TV watching duration**					
Never-<30 min	9 (3.3)	5 (55.6)	2 (22.2)	2 (22.2)	0.159^ⱡ^
30–2 h/day	149 (54.8)	99 (66.4)	41 (27.5)	9 (6.0)	
3–5 h/day	114 (41.9)	76 (66.7)	23 (20.2)	15 (13.2)	

^¥^Chi-square *p*-value, ^ⱡ^Fisher’s test *p*-value, PA, Physical activity; TV, Television; ^†^Heavy intensity exercise-running, aerobics, fast dancing, lifting heavyweight, playing football, or basketball, ^α^ Moderate intensity exercise-dancing, swimming.

Table 3 shows the relationship between the dietary habits of participants and body mass index status. The majority of the adolescents consumed three meals per day, while 21.0% skipped one of the daily meals. Breakfast was the most skipped meal by these adolescents (47.9%). About 25.7% of the adolescents sometimes took snacks, and these were mostly soft drinks (47.4%) and regular yoghurt (12.9%). About 59.6% of adolescents usually consume fast foods such as pizza, burgers, ice cream, etc. More than 3 in 10 (38.5%) adolescents consume fast foods one to three times a week. Adolescents who skipped supper (27.9%) were the most overweight or obese compared to those who skipped breakfast (23.3%) and lunch (18.8%) (*p* = 0.198). A higher proportion of adolescents who sometimes took soft drinks (27.9%) were overweight or obese (*p* = 0.476) than those who took other snacks. Adolescents who took fast foods daily (27.9%) had the highest proportions of overweight or obesity compared with those who took fast foods 1–3 times per week, 4–5 times per week, and those who took no fast foods (*p* = 0.456).

**TABLE 3:** Dietary habits and body mass index status.

		Body Mass Index status			*P*-value
			
Dietary habits (DH)	Total for DH	Normal	Overweight/obese	Underweight	
Number of meals/day	*N* = 272	*N* = 180	*N* = 66	*N* = 26	
Once	14 (5.1)	9 (64.3)	5 (35.7)	0 (0.0)	0.516 ^ⱡ^
Twice	55 (20.2)	36 (65.5)	13 (23.6)	6 (10.9)	
Thrice	181 (66.5)	118 (65.2)	43 (23.8)	20 (11.0)	
≥4	22 (8.1)	17 (77.3)	5 (22.7)	0 (0.0)	
**Skip meal**					
No	57 (21.0)	34 (59.6)	17 (29.8)	6 (10.5)	0.483^¥^
Yes	215 (79.0)	146 (67.9)	49 (22.8)	20 (9.3)	
**Meal skipped, for Yes, *N* = 215**					
Breakfast	103 (47.9)	74 (71.8)	24 (23.3)	5 (4.9)	0.198^¥^
Lunch	69 (32.1)	46 (66.7)	13 (18.8)	10 (14.5)	
Supper	43 (20.0)	26 (60.5)	12 (27.9)	5 (11.6)	
Sometimes take snacks					
Yes	70 (25.7)	49 (70.0)	18 (27.3)	3 (4.3)	0.220^ⱡ^
No	202 (74.3)	131 (64.9)	48 (23.8)	23 (11.4)	
**If snacking, which food**					
Soft drinks	129 (47.4)	81 (62.8)	36 (27.9)	12 (9.3)	0.476^ⱡ^
Fruits	32 (11.8)	25 (78.1)	3 (9.4)	4 (12.5)	
Cakes	12 (4.4)	7 (58.3)	3 (25.0)	2 (16.7)	
Yoghurt	35 (12.9)	22 (62.9)	8 (22.9)	5 (14.3)	
Others	17 (6.3)	11 (64.7)	4 (23.5)	2 (11.8)	
None	47 (17.3)	34 (72.3)	12 (25.5)	1 (2.1)	
**Usually took fast foods**					
No	110 (40.4)	70 (63.6)	30 (27.3)	10 (9.1)	0.634*^¥^*
Yes^α^	162 (59.6)	110 (67.9)	36 (22.2)	16 (9.9)	
**Number of times in a week for fast food**					
Daily	40 (16.2)	23 (57.5)	11 (27.5)	6 (15.0)	0.456^ⱡ^
1–3 times/Week	95 (38.5)	64 (67.3)	22 (23.2)	9 (9.5)	
4–5 times/Week	24 (9.7)	20 (83.4)	2 (8.3)	2 (8.3)	
None	88 (35.6)	59 (67.0)	22 (25.0)	7 (8.0)	

^¥^Chi-squared *p*-value, some cell count less than 5, so ^ⱡ^Fisher’s test *p*-value, DH, Dietary habit, ^α^ Fast foods included-pizza, chips, burgers, spicy fried chicken, potato chips, ice cream, overweight/obesity as BMI ≥ 25.0 Kg/m^2^.

Table 4 presents the relationship between calories, macronutrient intakes, and body mass index status. More adolescents who met the RDA for calories were overweight or obese (46.7%) than those who did not meet the RDA (23.0%) (*p* = 0.074). Similarly, a higher proportion of adolescents who met the RDA for dietary protein were overweight or obese (28.2%) than those who did not meet the RDA (22.5%) (*p* = 0.145). More adolescents who did not meet the RDA for dietary carbohydrates were slightly overweight or obese (25.0%) than those who met the RDA (24.3%) (*p* = 0.804).

**TABLE 4:** Calories, macronutrient intakes, and body mass index status.

		Body Mass Index status			*P*-value
			
Variable	RDA cut-off	Normal	Overweight/obese	Underweight	
Nutrient intakes		*N* = 180	*N* = 66	*N* = 26	
**Calories, Kcal**					
Met RDA		8 (53.3)	7 (46.7)	0 (0.0)	0.074^¥^
Not met RDA		172 (66.9)	59 (23.0)	26 (10.1)	
Mean calories		1685.9 ± 473.3	1804.9 ± 565.4	1570.8 ± 344.3	0.282**^†^**
Dietary carbohydrates, g	130 g				
Met RDA		177 (66.0)	65 (24.3)	26 (9.7)	0.804^¥^
Not met RDA		3 (75.0)	1 (25.0)	0 (0.0)	
Mean dietary carbohydrate		269.9 ± 76.6	283.4 ± 80.2	254.1 ± 57.1	0.376**^†^**
**Dietary protein, g**					
Met RDA		57 (67.1)	24 (28.2)	4 (4.7)	0.145^¥^
Not met RDA		123 (65.8)	42 (22.5)	22 (11.8)	
Mean dietary protein		42.7 ± 12.1	45.1 ± 13.6	40.3 ± 9.0	0.378**^†^**
**Dietary fibre, g**					
Met RDA		17 (73.9)	6 (26.1)	0 (0.0)	0.264^¥^
Not met RDA		163 (65.5)	60 (24.1)	26 (10.4)	
Mean dietary fibre		18.8 ± 6.7	19.0 ± 6.2	18.4 ± 4.7	0.302
Mean dietary fat, g		50.6 ± 18.4	57.0 ± 27.9	45.6 ± 12.6	0.950
**Anthropometry**					
Mean WC, cm		71.0 ± 6.2	82.3 ± 7.0	68.4 ± 20.5	**<0.001^†^**

Mean ± SD (standard deviation), ^†^Kruskal–Wallis test, Cells count less than 5, so ^¥^Fisher’s test *p*-value, RDA, Recommended dietary allowance; BMI, Body Mass Index (RDA cut-off). Bold values means the *p* values are significant.

Overweight/obese adolescents had the highest mean caloric intake (1804.9 ± 565.4 Kcal) compared with underweight (1570.8 ± 344.3 Kcal) and normal BMI adolescents (1685.9 ± 473.3 Kcal) (*p* = 0.282). Similarly, overweight/obese adolescents had the highest mean dietary fat intake (57.0 ± 27.9 g) compared with underweight adolescents (45.6 ± 12.6 g) and normal BMI adolescents (50.6 ± 18.4 g) (*p* = 0.950). Overweight/obese adolescents had the highest mean waist circumference (82.3 ± 7.0 cm) compared with underweight (68.4 ± 20.5 cm) and normal BMI adolescents (71.0 ± 6.2 cm) (*p* < 0.001).

Table 5 indicates Pearson correlation between calories, macronutrient intakes and anthropometric indices, when age and gender were adjusted. Independent of age and gender, caloric intake (*r* = 0.196, *p* = 0.001), dietary carbohydrate (*r* = 0.164, *p* = 0.007), protein (*r* = 0.160, *p* = 0.008), and fat (*r* = 0.215, *p* < 0.001) had weak, positive correlation with BMI. There was weak, positive correlation between calories (*r* = 0.156, *p* = 0.010), dietary carbohydrate (*r* = 0.142, *p* = 0.020), protein (*r* = 0.166, *p* = 0.006) and fat intakes (*r* = 0.142, *p* = 0.019), and waist circumference. There was a strong, positive correlation between waist circumference and BMI (*r* = 0.585, *p* < 0.001).

**TABLE 5:** Pearson correlation between calories, macronutrient intakes, and anthropometric indices.

	Anthropometric variable, r (*p*-value)	
	
Variables	WC, cm	BMI, Kg/m^2^
WC, cm	1	0.585 (**<0.001**)
Calories, Kcal	0.156 (**0.010**)	0.196 (**0.001**)
Dietary carbohydrates, g	0.142 (**0.020**)	0.164 (**0.007**)
Dietary protein, g	0.166 (**0.006**)	0.160 (**0.008**)
Dietary fat, g	0.142 (**0.019**)	0.215 (**<0.001**)
Dietary fibre, g	0.112 (0.066)	0.114 (0.060)

Adjusted for gender and age, r, Pearson correlation coefficient; WC, Waist circumference; BMI, Body mass index. Bold values means the *p* values are significant.

Table 6 presents the predictors of overweight and obesity among adolescents. Adolescent girls had 2.4 times higher odds of being overweight or obese compared with adolescent boys (adjusted odds ratio = 2.4, *p* = 0.094, 95% CI = 0.9–6.4). Adolescents who did not meet the RDA for calories had lower odds (unadjusted odds ratio = 0.3, *p* = 0.045, 95% CI = 0.1–0.9) of being overweight or obese compared with those who met the RDA. Adolescents who did not meet the RDA for dietary carbohydrates had higher odds (adjusted odds ratio = 1.1, *p* = 0.962, 95% CI = 0.1–10.5) of being overweight or obese compared with those who met the RDA. Adolescents who did not meet the RDA for dietary protein had reduced odds (unadjusted odds ratio = 0.7, *p* = 0.304, 95% CI = 0.4–1.3) of being overweight or obese compared with those who met the RDA.

**TABLE 6:** Predictors of overweight and obesity.

	Overweight/Obesity, BMI ≥ 25.0 Kg/m^2^					
	
Variable	UOR	*P*-value	95% CI	AOR	*P*-value	95% CI
**Gender**						
Adolescent boys	1.0			1.0		
Adolescent girls	2.5	0.068	0.9–6.7	2.4	0.094	0.9–6.4
**Calories, Kcal**						
Met RDA	1.0			1.0		
Not met RDA	0.3	**0.045**	0.1–0.9	0.4	0.081	0.1–1.1
**Dietary carbohydrate, g**						
Met RDA	1.0			1.0		
Not met RDA	1.0	0.972	0.1–10.2	1.1	0.962	0.1–10.5
**Dietary protein, g**						
Met RDA	1.0			1.0		
Not met RDA	0.7	0.304	0.4–1.3	0.9	0.778	0.5–1.7

Adjusted for the age of participants, UOR, Unadjusted odds ratio; AOR, adjusted odds ratio; CI, Confidence interval; RDA, Recommended dietary allowance. Bold values means the *p* values are significant.

## Discussion

The key findings were: inadequate caloric, dietary protein, and fibre intakes; inadequate recommended physical activity; participation in a sedentary lifestyle at home; poor dietary habits; and increased risk of overweight and obesity were common among the adolescents. Being an adolescent girl may be positively associated with the risk of obesity as well as, not consuming enough calories may be negatively associated with the risk of obesity among the adolescents.

In this study, the adolescents were more likely to perform high-intensity physical exercises at school, but while at home, they were more likely to live a sedentary lifestyle by watching long hours of television. About 90% of the adolescents were less likely to meet the WHO-recommended weekly 150 min of physical exercise. A previous study in Ghana reported that 75.4% of schooling adolescents aged 11–19 years in SHS had less physical activity ≥ 60 min per day (36). Rhodes et al. (37) also reported that 80% of school-aged adolescents did not meet the recommended daily physical exercise. The drivers of physical inactivity and sedentary behaviour among adolescents may be associated with affluent living and the use of communication technology (27, 38). In Ghana, adolescents perceived walking to school as an indicator of poverty, since it is common these days to see parents with high-income status driving their children to and from school (27). Also, adolescents in Ghana nowadays spend much time on the internet, computers, and other social media platforms rather than engaging in outdoor physical activities (27), leading to an increasingly sedentary lifestyle. The sedentary behaviour of adolescents poses a threat to our public health because of the associated risk of lifestyle diseases such as obesity, CVDs, and high blood pressure levels later in life. Also, findings revealed that adolescents with no physical exercise in the past week were more likely to be overweight or obese. Similarly, adolescents who watched television once to thrice per week were more likely to be overweight or obese. Although physical inactivity and sedentary lifestyle among these adolescents were not related to their current BMI status, other studies have shown that poor physical activity and sedentary lifestyle are associated with a risk of obesity (7, 39).

The study revealed that the adolescents were more likely to skip breakfast, and this was consistent with previous studies among schooling adolescents in Ghana (40, 41). Poor breakfast consumption among schooling adolescents in Ghana has been attributed to lack of time, desire to sleep longer, financial constraints, and lack of appetite in the morning (40, 41). The adolescents were more likely to consume soft drinks for snacks and frequently consume fast foods such as pizza, burgers, and ice cream. Similar studies in Ghana (8, 16) and Nigeria (42) have reported high consumption of sugar-sweetened beverages, sweets, and pastries as snacks among adolescents. The finding suggests that adolescents are developing unhealthy eating habits, which, if carried over into adulthood, may have a negative impact on their health. These fast foods contain few micronutrients but are high in calories, saturated and trans-fat, which when consumed may increase the risk of obesity and other NCDs (15). Beyond lifestyle diseases, fast food consumption is also associated with depression (43), and suicidal attempts (44). Some studies in Ghana and Nepal have reported that low socioeconomic status of parents (16), peer group and media influences (45), single parenting and junk foods available at home (46), in Nepal are the drivers of unhealthy eating behaviours among adolescents. Adolescents are fond of spending most of their time both in school and at home with peers and media activities, and these peers and media sometimes influence their choice of food (45). Additionally, the high influx of fast foods in the food environment may be the reason for unhealthy eating behaviour among adolescents (9). The dietary habits of adolescents were not related to their BMI status, and this was similar to findings by Annan et al. (47), who reported that breakfast consumption was not related to the BMI status of schoolchildren in Ghana. However, adolescents who ate fast food daily and those who sometimes drank soft drinks were more likely to be overweight or obese. Regular breakfast consumption was associated with a lower risk of obesity among adolescents (AOR = 0.6, 95% CI = 0.3–1.0) in a South African study (18), whereas skipping breakfast was associated with an increased risk of obesity (17).

In terms of nutrient intakes, the adolescents were less likely to meet the RDAs for calories, dietary protein, and dietary fibre but met the RDA for dietary carbohydrates. Inadequate caloric intake among adolescents has been reported in studies in Tanzania (48), and Zambia (49). The tendency of adolescents to underreport food intake might have contributed to the underestimation of energy intake (50). However, factors that affect their overall nutrient intake may include the kinds of foods available at home/school, the amount of time available to make food (51), knowledge of food content (52), and the ability to buy healthy snacks (53). Sociodemographic, behavioural, and environmental factors are also linked to different patterns of adolescent nutrition (54). Adolescent boys were more likely to consume higher calories, dietary carbohydrates, protein, fat, and fibre. A similar study in Tanzania found adolescent boys consumed higher dietary carbohydrates and protein than girls (48). Older adolescents were more likely to consume higher dietary fibre. The cause of the difference in nutrient intakes between genders and age groups is not fully understood and needs further exploration, especially the food sources and dietary patterns. However, differences have been attributed to the portion size consumed between adolescent boys and girls and age groups (48). Boys usually perform higher energy-demanding tasks than girls, so they may be more prone to the consumption of high-energy-dense foods, leading to higher caloric intake (55).

The study found the co-existence of undernutrition and overnutrition among these adolescents. Slightly lower than 10% of the adolescents were more likely to be underweight, while one in four (24.3%) adolescents were more likely to be either overweight or obese. The proportion of overweight and obese adolescents in our study is far lower than the 35 and 47.0% overweight and obese prevalence among adolescents in South Africa (18) and Ghana (56). Our finding is higher than the 17% overweight/obesity prevalence among 9–15-year-old school children in urban Ghana (57). The high prevalence of overweight and obesity is worrying as these adolescents may be at risk of adult obesity and, consequently, may lead to a risk of lifestyle-related diseases such as CVDs, high blood pressure, and diabetes. An increased overweight or obesity prevalence among adolescents highlights the necessity for regular surveys, growth monitoring, and effective prevention strategies. For instance, nutrition and physical activity education interventions are reported to reduce the BMI-for-age status of schoolchildren aged 9–13 years in Ghana (58). The study found that an increase in calories, dietary carbohydrates, protein, and fat intakes could be associated with an increase in BMI and WC levels. We found that an increase in waist circumference was strongly associated with an increase in BMI. In the regression analysis, the adolescent girls were more likely to be overweight or obese (AOR = 2.4, *p* = 0.094, 95% CI = 0.9–6.4). A similar finding by Debaila et al. (18) showed that adolescent girls had increased odds (AOR = 2.9, 95% CI = 1.7–4.8) of being overweight or obese. The younger adolescents were more likely to be overweight or obese. Adolescents who did not meet RDA for calories had reduced odds (UOR = 0.3, *p* = 0.045, 95% CI = 0.1–0.9) of being overweight or obese, implying that low caloric intake may reduce obesity risk. We are aware that the cross-sectional design of the study limits the interpretation of the findings; however, this study has given insight and relevant information on dietary behaviour, physical activity, sedentary behaviour, nutrient intake, and obesity prevalence among these understudied populations in Ghana, for stakeholders to prioritize interventions tailored to suit this target population.

## Conclusion

Poor dietary habits and intake and a sedentary lifestyle were observed among the schooling adolescents. Overweight and obesity are still prevalent among adolescents in Ghana. Being an adolescent girl was associated with obesity risk, while adolescents who did not meet caloric intake had a protective effect against the risk of being overweight or obese. An efficient and effective nutritional and lifestyle education programme should be promoted in the communities to adopt a healthy lifestyle among adolescents in rural and peri-urban communities in Ho municipality and the country at large.

## Data availability statement

The raw data supporting the conclusions of this article will be made available by the authors, without undue reservation.

## Ethics statement

The studies involving human participants were reviewed and approved by Committee on Human Research Publication and Ethics, the School of Medicine and Dentistry, Kwame Nkrumah University of Science and Technology. Written informed consent to participate in this study was provided by the participants’ legal guardian/next of kin.

## Author contributions

SA, KN, and MT: conceptualization of study, study design, and methodology. SA, MT, and OA-B: formal data analysis. SA and VA: writing—original first draft manuscript preparation. OA-B: review and second draft manuscript. MT and OA-B: writing—review and editing. All authors read and approved submitted manuscript.
